# The Lived Experience of Managing Diabetes and Hypertension: A Qualitative Study From a Hilly Region in Uttarakhand, India

**DOI:** 10.7759/cureus.97128

**Published:** 2025-11-17

**Authors:** Santosh Kumar, Meenakshi Khapre, Ekta D Pathak, Sakshi Gautam

**Affiliations:** 1 Family and Community Medicine, All India Institute of Medical Sciences, Rishikesh, Rishikesh, IND; 2 Community Medicine, All India Institute of Medical Sciences, Rishikesh, Rishikesh, IND; 3 Public Health, All India Institute of Medical Sciences, Rishikesh, Rishikesh, IND

**Keywords:** diabetes, hypertension, perception, rural area, self-management practice

## Abstract

Background

Diabetes and hypertension are significant public health concerns in India, particularly in rural communities where access to healthcare and health literacy may be limited. This study explores the lived experiences of individuals managing these chronic conditions in a rural Indian setting. This study aimed to understand the perceptions, knowledge, self-management practices, and challenges faced by individuals living with diabetes and hypertension in a rural Indian community.

Methodology

This phenomenological qualitative research study was conducted using semi-structured interviews with 40 participants (20 with diabetes, 20 with hypertension) from selected villages of Tehri district, Uttarakhand. In-depth interviews were conducted. Audio tapes of each interview were made. The native language (Garhwali) tape was verbatim translated into English after completion. Data were analyzed using thematic analysis to identify recurring patterns and themes.

Results

The study revealed several key themes, including a limited understanding of disease etiology and risk factors, the perceived severity and potential complications of the conditions, diverse medication adherence behaviors, the use of alternative medicine, and significant barriers to effective self-management. Participants frequently cited misinformation about disease and its management, unaffordability of healthy food options, lack of accessibility of health facilities, and lack of self-management guidance from their physicians as key challenges to managing their condition.

Conclusions

This study reveals the perception and understanding of a hilly and rural population about diabetes and hypertension in terms of self-management practices and challenges. Some of the participants were well aware of the life-threatening complications of diabetes, with some having misconceptions about causes and risk factors. Their belief about medications, social practices, and concerns for diabetes brought a new dimension to explore in the prevention and control of these diseases in rural India.

## Introduction

Non-communicable diseases (NCDs) have become the most prevalent and expensive health issue in the world, contributing 68% of mortality globally and 60% of all deaths in India. As a result of the recent epidemiological shift, chronic disease has become more prevalent in low- and middle-income nations, where 86% of early deaths from chronic disease occur. India is also undergoing this rapid transformation in the form of rapid urbanization, which leads to economic rise and improved living standards, but personal behavior, lifestyle, and environmental exposure become potential risks [1]. This resulting NCD burden has overtaken the burden of communicable diseases such as water- or vector-borne diseases. Diabetes and hypertension have emerged as the most prevalent NCDs in recent years.

The number of people with diabetes in India is expected to rise to an astonishing 134 million by 2045 [2]. Similarly, there are expected to be 214 million people with hypertension by 2030 [3]. In addition, both problems are significant factors that contribute to the development of major cardiovascular diseases, such as coronary heart disease and stroke, which are widespread and a major cause of fatalities from NCDs [3].

NCDs threaten progress toward the 2030 Agenda for Sustainable Development, which includes a target of reducing the probability of death from NCDs between ages 30 and 70 years by one-third by 2030 [1]. However, the detection and treatment rates for both conditions are appallingly low in the country, particularly in rural areas. A meta-analysis of hypertension across India noted that only 25.3% of rural hypertensive patients were aware of their diagnosis and were being treated, with control being achieved in only one-tenth of this population [4]. Another study found that only 49.3% of patients took antidiabetic drugs, and fewer than 25% of diabetics engaged in self-care activities [5].

A balanced diet, healthy lifestyle, illness awareness, treatment adherence, and routine follow-up are all crucial in addressing and preventing complications associated with hypertension and diabetes mellitus. However, the lack of affordable and accessible healthcare in India is a well-known barrier, with the poorest and rural people frequently being the most affected [6]. Thus, patients who come from longer distances are entirely left on their own to understand their disease process and navigate through confusing self-management pathways of disease [7]. Studies have shown a lack of awareness regarding target levels, causes, complications, and self-management of illnesses, and several myths associated with their causes, resulting in poor adherence to self-management practices. However, most of these studies are conducted in the southern or western part of India [7-10], reflecting a paucity of studies from the northern hilly areas. Factors such as poverty, limited education, inadequate infrastructure, and cultural beliefs contribute to the challenges of managing chronic conditions such as diabetes and hypertension. This study addresses the need for an in-depth understanding of the experiences of how the chronic physical, social, and environmental conditions transform into diabetes and hypertension in individuals living in a specific rural Indian setting.

This study seeks to answer the following research question: What are the lived experiences of individuals managing diabetes and hypertension in a rural Indian community? The study aimed to explore participants’ understanding of the causes, risk factors, and complications of diabetes and hypertension; to describe the self-management practices adopted by participants, including medication adherence, dietary modifications, and physical activity; identify the challenges and barriers faced by participants in managing their conditions; and examine the role of cultural beliefs, social support, and healthcare access in shaping participants’ experiences.

## Materials and methods

Study setting

This qualitative research was conducted in the Tehri district of hilly Uttarakhand state, located in the northern part of India. Lying in the western Himalayas, Tehri is well known for its scenic splendor and the Tehri Dam, the highest and largest dam in Asia and the 10th tallest dam in the world. The district has a population of 618,931 inhabitants, with more females (320,945) than males (297,986), and a density of 169 persons/km. According to the 2011 census, 90.5% of the population speaks Garhwali as their primary language in the district [11].

Study design, participants, and data collection

An exploratory, phenomenological, qualitative study was conducted under the project Title “Doorstep Primary Care Model’ from March to August 2022 in Tehri Garhwal, Uttarakhand, among adults aged over 30 years with diabetes and hypertension selected using purposive sampling.

For the study, a total of 40 subjects were interviewed in-depth utilizing a pre-designed and pre-tested guide. The interview guide covered topics such as disease perception, knowledge of risk factors and complications, self-management practices, challenges faced, and sources of support. Interviews lasted for 45-60 minutes and were audio-recorded with participants’ consent. The in-depth interviews were conducted at the patient’s convenience in the local language. The transcript was prepared by field staff under the supervision of the co-investigator of the project. Field notes were also prepared to mention expressions of participants, and verbatim transcripts were prepared by the second author present at the time of the in-depth interview. An interview was conducted twice during observation of the community for six months. The second author conducted the in-depth interview.

Sample size calculation

Sample size was determined by information power and iterative assessment of thematic saturation rather than statistical calculation. We planned a target of 40 in-depth interviews through a purposive sampling method (approximately 20 people with diabetes and 20 with hypertension) based on the study’s focused aims. Data collection proceeded in blocks, with a pre-specified saturation grid used to document the emergence of new codes. Recruitment ceased in each stratum when two consecutive interview blocks produced no new themes or substantive meanings relevant to the objectives. The same participant was interviewed on two separate occasions to clarify responses, explore new insights, or ensure completeness of data. These procedures are consistent with best practices in qualitative health research and are fully auditable.

We planned to speak with around 40 individuals living with diabetes and hypertension (about 20 in each group). This number was chosen because it allowed us to capture diverse voices across gender, age, and social background, while still keeping the dataset manageable for in-depth analysis.

Design of the interview guide

We designed the interview guide with the basic concept of the health belief model and the self-management framework, which comprises the perception of risk, severity, and barrier. While structuring the interview guide, we covered various domains of people’s perceptions, needs, and challenges. These included understanding the condition, perceived severity and complication, self-management complication, adherence and poor compliance, barrier, challenges, and support system. All the questions were kept open-ended, culturally sensitive, and non-judgmental. Probes were added to encourage thick description, language was kept simplified, and later translated into the local dialect for accessibility.

Validation of the interview guide

Content validity was ensured by expert review and alignment with study objectives and theoretical frameworks. Face validity was established by testing with community participants for comprehensibility. Construct validity was assessed by checking whether questions elicited responses across the intended domains (e.g., perceptions, practices, barriers). Cultural validation was achieved through local translation, back-translation, and community advisory input to confirm appropriateness of terms and metaphors. For ongoing iterative validation, during early interviews, the research team met weekly to assess whether questions generated rich, relevant data. Probes were adapted in real time (reflexive validation). For saturation monitoring, thematic saturation was tracked using a saturation grid, ensuring adequacy of data coverage for each domain.

Participant recruitment and characteristics

Participants were recruited through purposive sampling from a local community health center. Inclusion criteria were (1) diagnosis of type 2 diabetes or hypertension, (2) residence in the study area for at least one year, and (3) willingness to participate in the study. A total of 40 participants were recruited (20 with diabetes, 20 with hypertension), ensuring diversity in age, gender, education level, and socioeconomic status.

Data analysis approach and quality assurance measures

The audio-recorded interviews were transcribed verbatim and translated into English. Gemini AI was used to frame the interview guide. The data were analyzed manually using thematic analysis following Braun and Clarke’s (2006) [12] framework. This involved (1) familiarization with the data, (2) generating initial codes, (3) searching for themes, (4) reviewing themes, (5) defining and naming themes, and (6) producing the report.

Data saturation was reached when no new themes emerged from the interviews. Quality assurance measures included (1) member checking (returning transcripts to participants for verification), (2) peer debriefing (discussing coding and interpretation with other researchers), and (3) maintaining a reflexive journal to document the researchers’ biases and assumptions.

Ethical considerations

The study received approval from the Institutional Ethical Council, All India Institute for Medical Sciences, Rishikesh (approval number: AIIMS/IEC/21/576). The interviewees were briefed on the study’s objectives before the interview, and they were made aware that their participation was voluntary and that they could choose to opt out at any point. Each interviewee provided their written consent. Throughout the interview, the interviewees’ privacy and confidentiality were upheld.

## Results

**Figure 1:**
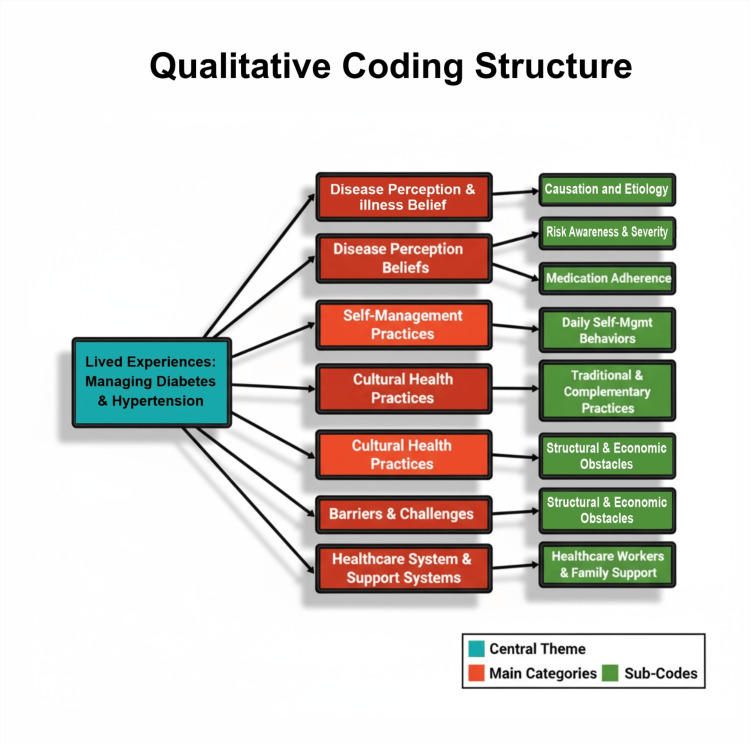
Qualitative coding structure.

The thematic analysis revealed several key themes related to the lived experiences of individuals managing diabetes and hypertension in the rural Indian community. A summary table of themes, a code frequency table, and a conceptual model illustrating theme relationships are provided in Table 1, Table 2, and Figure 1.

**Table 1:** Summary of themes.

Theme	Key characteristics	Representative wuote
Limited disease understanding	Misconceptions about causes, risk factors, and complications; lack of awareness about lifestyle modifications	“I have high or low blood sugar because of certain food items or when I drink alcohol with someone once a week. So, I think it may be due to fault in food habits.” (Participant 8)
Perceived severity and complications	Recognition of the potential dangers of uncontrolled disease; fear of long-term complications affecting vital organs	“Sugar is very dangerous; it completely destroys the whole body. It can result in other diseases too. Our eyes, kidneys, heart, and liver can be affected by this.” (Participant 15)
Diverse medication adherence	Varied patterns of medication adherence; factors influencing adherence, including beliefs about medication, side effects, and access to healthcare	“Initially, I took medicine whenever I felt my blood pressure rising. Then, the doctor said to take it regularly, otherwise a heart attack may occur if not controlled. So, now I take it regularly.” (Participant 23) “I don’t take medicines, I only take herbs because if I will start taking medicine, I will have to take it regularly, my body will become habitual of it. So, I avoid it.” (Participant 18)
Traditional and complementary medicine use	Reliance on traditional remedies, herbal medicines, and alternative therapies; perceived benefits and cultural significance of traditional practices	“I take ayurvedic medicine, Himalaya’s which is under my BP control. I never take any other medication by doctors or any other home remedies.” (Participant 19)
Barriers to self-management	Challenges related to affordability, accessibility, cultural beliefs, and social support; impact on lifestyle modifications and healthcare-seeking behaviors	“See, they say to eat this and that, but in villages all these things cannot be met. Like they say to eat Jhangora (millets) is beneficial, but it is so costly around 80-90 rs per kg, how unemployed people like us will eat and who will buy?” (Participant 32)
Influence of the healthcare provider	Impact of the healthcare providers and counselors on patients’ self-management and medication adherence	” I eat chapatis made up of manduwa (Finger millet) and barnyard millet as suggested by the doctor. Also, sweets are completely prohibited. Initially, I was not able to avoid it, but now it has become a habit. I also took my medicines regularly.” (Participant 22)

**Table 2:** Code frequency table.

Code	Diabetes (N = 20)	Hypertension (N = 20)	Total (N = 40)
Misconceptions about causes	15	12	27
Fear of complications	18	16	34
Adherence to medication	12	14	26
Use of traditional remedies	10	8	18
Financial barriers	16	14	30
Accessibility barriers	11	10	21
Lack of self-management guidance from doctors	12	9	21

Theme 1: Limited disease understanding

Definition

This theme reflects participants’ lack of comprehensive knowledge about the causes, risk factors, and complications of diabetes and hypertension.

Description

Many participants demonstrated limited understanding of the underlying causes of their conditions. They often attributed diabetes to excessive consumption of sweets or tea, while hypertension was primarily linked to stress or anger. There was little recognition of risk factors such as obesity, physical inactivity, or family history. This limited understanding contributed to misconceptions about self-management practices.

“It may have happened because of stress/tension or excessive tea intake. As we are shopkeepers, we drink at least 8-10 cups a day.” (Participant 9, diabetes).

Internal Variations and Sub-themes

Misattribution: Incorrect association of diseases with typhoid fever or weather change.

Superficial knowledge: Knowing only a few symptoms of the disease.

Connections to Other Themes

This theme directly influences self-management practices, as individuals with limited knowledge may not understand the importance of lifestyle modifications or medication adherence.

Contradictory Evidence and Outlying Cases

While the majority displayed knowledge gaps, a few participants who had received extensive health education demonstrated a more accurate understanding of their conditions.

Theme 2: Perceived severity and complications

Definition

This theme captures participants’ perception of diabetes and hypertension as serious and potentially life-threatening conditions, along with their awareness of the long-term complications associated with uncontrolled disease.

Description

Despite limited knowledge about the causes and risk factors, many participants expressed a strong awareness of the potential severity of diabetes and hypertension. They recognized that uncontrolled blood sugar and blood pressure could lead to serious complications affecting vital organs such as the eyes, kidneys, heart, and brain. This perception of severity often motivated participants to seek treatment and adhere to medical advice, to a certain extent.

“Sugar is very dangerous; it completely destroys the whole body. It can result in other diseases too. Our eyes, kidneys, heart, and liver can be affected by this.” (Participant 15, diabetes) (Table 1).

Internal Variations and Sub-themes

Fear of amputation: High anxiety surrounding the possibility of limb amputation.

Visual impairment concerns: Intense fear about losing eyesight.

Connections to Other Themes

This theme influences medication adherence and self-management practices. The fear of complications motivates some participants to adhere to treatment regimens, while others may be overwhelmed by the perceived severity of the disease.

Contradictory Evidence and Outlying Cases

Some participants downplayed the severity of their conditions, particularly if they were asymptomatic or had experienced minimal complications.

Theme 3: Diverse medication adherence

Definition

This theme encompasses the varied patterns of medication adherence observed among participants, along with the factors influencing their adherence behaviors.

Description

Medication adherence was not uniform across participants. Some individuals diligently followed their prescribed medication regimens, while others were less consistent or preferred to use alternative therapies. Factors influencing adherence included beliefs about medication efficacy, side effects, access to healthcare, and perceived severity of the disease.

“Initially, I took medicine whenever I felt my blood pressure rising. Then, the doctor said to take it regularly, otherwise a heart attack may occur if not controlled. So, now I take it regularly.” (Participant 23, hypertension).

“I don’t take medicines, I only take herbs because if I will start taking medicine, I will have to take it regularly, my body will become habitual of it. So, I avoid it.” (Participant 18, diabetes) (Table 1).

Internal Variations and Sub-themes

Intentional non-adherence: Actively choosing not to follow medical advice.

Unintentional non-adherence: Forgetting doses or running out of medication due to logistical barriers.

Connections to Other Themes

This theme is influenced by disease understanding, perceived severity, cultural beliefs, and access to healthcare.

Contradictory Evidence and Outlying Cases

There were instances where participant knowledge of the disease and its management was good, but adherence was poor due to factors such as financial difficulties, accessibility issues, or cultural beliefs.

Theme 4: Traditional and complementary medicine use

Definition

This theme highlights the reliance on traditional remedies, herbal medicines, and alternative therapies for managing diabetes and hypertension, along with the perceived benefits and cultural significance of these practices.

Description

Many participants reported using traditional and complementary medicine (T&CM) practices alongside or in place of conventional medical treatment. These practices included herbal remedies, Ayurvedic medicines, yoga, and dietary modifications based on traditional beliefs. Participants often perceived T&CM as natural, safe, and culturally appropriate alternatives to pharmaceutical medications.

“I take ayurvedic medicine, Himalaya’s which is under my BP control. I never take any other medication by doctors or any other home remedies.” (Participant 19, hypertension).

Internal Variations and Sub-themes

Integration of T&CM: Using both conventional and traditional treatments.

Exclusive reliance on T&CM: Rejecting conventional medicine in favor of traditional practices.

Connections to Other Themes

This theme is closely linked to cultural beliefs, disease understanding, and access to healthcare.

Contradictory Evidence and Outlying Cases

Although many participants used TCM alongside modern medicine, some individuals knew the effectiveness of medicines, but due to a lack of accessibility, affordability, or fear of getting addicted to medicines, they shifted to T&CM.

Theme 5: Barriers to self-management

Definition

This theme identifies the challenges and barriers faced by participants in managing their diabetes and hypertension, including affordability, accessibility, cultural beliefs, and social support.

Description

Participants encountered numerous barriers to effective self-management. Affordability was a major concern, as healthy foods (such as millets) and medications were often perceived as too expensive. Access to healthcare services was also a challenge, particularly for those living in remote areas. Cultural beliefs and social support systems also played a role in shaping self-management practices.

“See, they say to eat this and that, but in villages all these things cannot be met. Like they say to eat Jhangora (millets) is beneficial, but it is so costly around 80-90 rs per kg, how unemployed people like us will eat and who will buy?” (Participant 32, diabetes).

Internal Variations and Sub-themes

Financial constraints: Inability to afford medications, healthy foods, or transportation to healthcare facilities.

Geographic isolation: Difficulty accessing healthcare services due to distance and lack of transportation.

Sociocultural influences: Non-compliance due to family beliefs, dietary preferences, and restrictions due to social functions.

Connections to Other Themes

This theme is influenced by socioeconomic status, cultural context, and healthcare infrastructure.

Contradictory Evidence and Outlying Cases

Some participants with financial resources and strong social support systems were able to overcome these barriers and successfully manage their conditions.

Theme 6: Influence of the healthcare provider

Definition

This theme identifies the effect that the healthcare provider has on encouraging patients to manage their disease and adhere to medications in rural settings.

Description

Participants were more likely to follow medical prescriptions and manage their disease if healthcare providers and counselors played a role in educating and guiding patients about the management of diabetes and hypertension.

“I eat chapatis made up of manduwa (Finger millet) and barnyard millet as suggested by the doctor. Also, sweets are completely prohibited. Initially, I was not able to avoid it, but now it has become a habit. I also took my medicines regularly” (Participant 22, hypertension).

Internal Variations and Sub-themes

Lack of guidance: Participants expressed receiving little or no guidance from doctors about self-management of the disease.

Healthcare provider’s encouragement: Positive influence due to guidance and motivation from healthcare providers.

Connections to Other Themes

This theme directly influences self-management practices, as individuals with proper education are more likely to follow medical advice and make informed decisions about their health.

Contradictory Evidence and Outlying Cases

There were instances where participants faced a communication gap with healthcare providers, impacting their willingness to adhere to medications.

## Discussion

This study investigated the perspectives, experiences, and barriers to and enablers of self-management of diabetes and hypertension in the hilly areas of Uttarakhand. Self-care for diabetes entails leading a healthy lifestyle alongside taking recommended medications and getting regular tests. Overall, participants’ knowledge was conflicting, especially when it came to the causes and complications of the conditions. Although most participants seemed to be aware of the rise in prevalence in their communities, very few participants were able to link behavioral risk factors such as diet, alcohol, smoking, and stress to this rise. These findings were similar to a study conducted in rural South India, which indicated unawareness of target levels and complications of diabetes [8]. According to research evidence, those lacking appropriate knowledge about the disease, risk factors, and treatment adherence are more likely to have poor control levels. Thus, patients require proper knowledge of factors associated with the biological control of diabetes and hypertension to achieve better management and avoid complications [13].

It is crucial to recognize and discuss sensitive religious and cultural issues with patients and their families. Moreover, family education can help improve adherence among patients. Similar barriers were reported in studies conducted in Bangladesh, Pakistan, and Iraq [14-16]. To control the disease’s progression, physical activity is pivotal for NCD patients. However, older age and comorbidities were reported as frequent barriers [17]. Similar to Bukhsh et al. [14], several myths were also observed, such as respondents (especially housewives) thinking that their household work was a fair replacement for exercise.

The results of our study indicated that medication adherence was the most widely used method of diabetes self-management. This might be due to the fact that taking medications consistently is simpler than following other self-management strategies. However, being far from a medical facility frequently results in interruptions to a patient’s medication schedule. This is consistent with a systematic review that reported that traveling a greater distance results in indirect costs for travel and income losses due to the inconvenient times required for the consultation, which often discourage patients [18]. On the other hand, the results show that many patients resorted to natural remedies. These natural remedies were either taken in addition to or in place of antidiabetic or antihypertensive drugs. However, these medications cause more harm than good depending on dosage, frequency, and duration of use. Damnjanovic et al. [19] reported that diabetic respondents who used herbal dietary supplements reported more frequent symptoms of hypoglycemia than their counterparts. Similarly, combining various herbal remedies such as garlic with prescription medications for hypertension may have unintended consequences [20].

Participants who receive counseling from healthcare professionals can better manage their diseases. The participants had varying levels of experience with healthcare professionals. They reported that encouragement, risk communication, and education provided by healthcare professionals encouraged them to increase their adherence to their medications, physical activity levels, and adoption of healthy eating habits. These results are in line with several studies, which showed that participants’ lifestyles can be improved and their disease can be managed more effectively through the provision of education and assurance from healthcare professionals [14,18,21].

In chronic illnesses, perceptions of the illness can create a supportive environment that encourages self-care. Study participants followed their treatment plans because of the fear of complications brought on by poorly managed diabetes and hypertension. This corroborates with findings of Kanungo et al. [22], who found that people who felt their condition was serious visited qualified medical professionals, particularly in the private sector, which probably helped them get past any obstacles that might have prevented them from receiving better healthcare (possible obstacles included cost, transportation, availability, and waiting times).

The results of our study have some practical ramifications for rural India’s healthcare system. First, healthcare professionals must inform their patients and family members about the significance of a healthy diet and regular exercise due to the lack of knowledge in this area. Second, self-care education must be tailored to meet the individual patient’s needs and include relevant information about the causal factors, complications, and prognosis of diabetes. Third, government initiatives need to focus on the provision of comprehensive outpatient care, which includes the availability of specialists, trained NCD educators, a consistent supply of medications, and essential laboratory investigation in rural settings.

Limitations

Although our study provides fresh perspectives into the practices and experiences of rural hypertension and diabetes patients, it does have a few limitations. One of the limitations of a qualitative study is the potential for selection bias. Second, self-care practices were not examined in relation to the educational backgrounds of study participants. Furthermore, the study did not seek the perspectives of healthcare providers, which could have provided a more comprehensive picture of the problem. However, it is crucial to acknowledge that the qualitative design places an emphasis on problem-specific analysis rather than generalizability.

## Conclusions

Overall, our study noted that people’s perceptions of, experiences with, and management of their own health are affected by their knowledge of the causation pathway and consequences of the disease. This study reveals the perception and understanding of the hilly and rural population about diabetes and hypertension in terms of self-management practices, cultural practices, and affordability. Some of the participants were well aware of the life-threatening complications of diabetes, but some had misconceptions about the cause and risk factors of hypertension and diabetes. Affordability and accessibility were major limitations observed in this study due to the difficult geographical terrain. The inclination of villagers for alternative medicine or home-based treatment may be due to poor affordability and availability of allopathic medicine. Their belief about medications, social practices, and concerns for diabetes brought a new dimension to explore in the prevention and control of this disease in rural India. This study recommends advocating culturally tailored health education programs that address misconceptions about diabetes and hypertension, promote healthy lifestyle choices, and empower individuals to take control of their health. These programs should be delivered in local languages and incorporate visual aids and interactive methods to enhance understanding.

## Appendices

**Table 3:** Interview guide.

Section 1 — Background information
Age group (30–40, 41–50, 51–60, 61+)
Gender
Education level
Occupation/livelihood
Years since diagnosis (diabetes/hypertension)
Section 2 — In-depth interview questions
Q1. Understanding of condition (to explore disease perceptions)
What do you think caused your condition (diabetes/hypertension)?
What risk factors or lifestyle habits do you believe affect it?
How do people in your community usually explain this illness?
Q2. Perceived severity and complications (to capture awareness and fears)
How serious do you feel your condition is?
What complications or effects on the body do you know about?
What examples from family/community influenced your view?
Q3. Self-management practices (to describe daily management)
What steps do you take to control your condition (medicines, diet, activity)?
How do you remember to take medicines or follow routines?
Can you share a day when it was especially hard or easy?
Q4. Medication adherence (to understand behaviors and barriers)
How do you decide when to take or skip medicines?
Have you ever changed your medication routine on your own?
What makes it easier or harder to continue taking medicines regularly?
Q5. Traditional and complementary practices (to explore cultural influences)
Do you use any traditional remedies or local treatments?
What benefits do you see from these practices?
How do you combine them with modern medicines, if at all?
Q6. Barriers and challenges (to identify obstacles)
What difficulties do you face in managing your illness (cost, distance, family beliefs)?
How does the availability of food, medicine, or transport affect you?
Can you describe a moment when these barriers made it hard to cope?
Q7. Role of healthcare providers and support systems (to explore external support)
What kind of advice or support do you get from doctors, ASHAs, or ANMs?
How helpful have these interactions been for you?
What kind of support do you receive from family or community?

## References

[1] Nethan S, Sinha D, Mehrotra R (2017). Non communicable disease risk factors and their trends in India. Asian Pac J Cancer Prev.

[2] Oberoi S, Kansra P (2020). Economic menace of diabetes in India: a systematic review. Int J Diabetes Dev Ctries.

[3] Mohan S, Jarhyan P, Ghosh S (2018). UDAY: a comprehensive diabetes and hypertension prevention and management program in India. BMJ Open.

[4] Anchala R, Kannuri NK, Pant H, Khan H, Franco OH, Di Angelantonio E, Prabhakaran D (2014). Hypertension in India: a systematic review and meta-analysis of prevalence, awareness, and control of hypertension. J Hypertens.

[5] S A, T M (2014). Self care and medication adherence among type 2 diabetics in Puducherry, southern India: a hospital based study. J Clin Diagn Res.

[6] Chang H, Hawley NL, Kalyesubula R, Siddharthan T, Checkley W, Knauf F, Rabin TL (2019). Challenges to hypertension and diabetes management in rural Uganda: a qualitative study with patients, village health team members, and health care professionals. Int J Equity Health.

[7] Lall D, Engel N, Devadasan N, Horstman K, Criel B (2019). Challenges in primary care for diabetes and hypertension: an observational study of the Kolar district in rural India. BMC Health Serv Res.

[8] Anitha Rani M, Shriraam V (2019). Are patients with type 2 diabetes not aware or are they unable to practice self-care? A qualitative study in rural South India. J Prim Care Community Health.

[9] Matpady P, Maiya AG, Saraswat PP, Mayya SS, Pai MS, S AD, Umakanth S (2020). Dietary self-management practices among persons with T2DM: an exploratory qualitative study from western-coast of India. Diabetes Metab Syndr.

[10] Newtonraj A, Arun S, Bazroy J, Tovia S (2017). Lay perspectives on causes and complications of hypertension; and barrier to access health care by known hypertensive patients: a qualitative study from a rural area of South India. Int J Community Med Public Health.

[11] (2023). Government of Uttarakhand. District Tehri Garhwal. https://tehri.nic.in/.

[12] Braun V, Clarke V (2006). Using thematic analysis in psychology. Qual Res Psychol.

[13] Dey S, Mukherjee A, Pati MK (2022). Socio-demographic, behavioural and clinical factors influencing control of diabetes and hypertension in urban Mysore, South India: a mixed-method study conducted in 2018. Arch Public Health.

[14] Bukhsh A, Goh BH, Zimbudzi E, Lo C, Zoungas S, Chan KG, Khan TM (2020). Type 2 diabetes patients' perspectives, experiences, and barriers toward diabetes-related self-care: a qualitative study from Pakistan. Front Endocrinol (Lausanne).

[15] Mikhael EM, Hassali MA, Hussain SA, Shawky N (2019). Self-management knowledge and practice of type 2 diabetes mellitus patients in Baghdad, Iraq: a qualitative study. Diabetes Metab Syndr Obes.

[16] Kabir A, Karim MN, Billah B (2022). Health system challenges and opportunities in organizing non-communicable diseases services delivery at primary healthcare level in Bangladesh: a qualitative study. Front Public Health.

[17] Lidegaard LP, Schwennesen N, Willaing I, Faerch K (2016). Barriers to and motivators for physical activity among people with type 2 diabetes: patients' perspectives. Diabet Med.

[18] Krishnamoorthy Y, Rajaa S, Rehman T, Thulasingam M (2022). Patient and provider's perspective on barriers and facilitators for medication adherence among adult patients with cardiovascular diseases and diabetes mellitus in India: a qualitative evidence synthesis. BMJ Open.

[19] Damnjanovic I, Kitic D, Stefanovic N, Zlatkovic-Guberinic S, Catic-Djordjevic A, Velickovic-Radovanovic R (2015). Herbal self-medication use in patients with diabetes mellitus type 2. Turk J Med Sci.

[20] Rahmawati R, Bajorek BV (2017). Self-medication among people living with hypertension: a review. Fam Pract.

[21] Basu S, Sharma N (2019). Diabetes self-care in primary health facilities in India - challenges and the way forward. World J Diabetes.

[22] Kanungo S, Bhowmik K, Mahapatra T, Mahapatra S, Bhadra UK, Sarkar K (2015). Perceived morbidity, healthcare-seeking behavior and their determinants in a poor-resource setting: observation from India. PLoS One.

